# Clinical value of Lp-PLA2, LDL-C, HDL-C, hs-CRP, leukocyte, FPG and HbA1c in type 2 diabetes mellitus patients with acute ischemic stroke

**DOI:** 10.3389/fendo.2025.1546961

**Published:** 2025-04-17

**Authors:** Huimin Shen, Anyan Huang, Bingxin Wang, Xiaohua Huang, Ruiqian Chen

**Affiliations:** ^1^ Department of Brain Center, People’s Hospital of Chenghai District, Shantou, Guangdong, China; ^2^ Department of Health Care, Shantou Maternal and Child Health Hospital, Shantou, Guangdong, China; ^3^ Department of Pediatrics, the Second Affiliated Hospital of Shantou University Medical College, Shantou, Guangdong, China; ^4^ Department of Information, the Second Affiliated Hospital of Shantou University Medical College, Shantou, Guangdong, China

**Keywords:** human plasma lipoprotein-associated phospholipase A2, atherosclerotic, type 2 diabetes mellitus, acute ischemic stroke, clinical evaluation

## Abstract

**Background:**

To evaluate the clinical significance of human plasma lipoprotein-associated phospholipase A2 (Lp-PLA2), low-density lipoprotein cholesterol (LDL-C), high-density lipoprotein cholesterol (HDL-C), high-sensitivity C-reactive protein (hs-CRP), leukocyte count, fasting plasma glucose (FPG), and glycated hemoglobin (HbA1c) in patients with Type 2 diabetes mellitus (T2DM) and acute ischemic stroke (AIS).

**Methods:**

A total of 155 T2DM patients with AIS, admitted to the Second Affiliated Hospital of Shantou University Medical College between October 2023 and October 2024, were included in the stroke group. Additionally, 86 T2DM subjects from the same period were included in the T2DM control group. Serum levels of Lp-PLA2, LDL-C, HDL-C, hs-CRP, leukocyte count, FPG, and HbA1c were compared between the T2DM-AIS group and the T2DM control group, as well as among T2DM-AIS patients with different infarct sizes and degrees of neurological impairment. The clinical value of the above indexes in the diagnosis of T2DM with AIS was analyzed by receiver operating characteristic curve (ROC curve).

**Results:**

Serum levels of Lp-PLA2 (142.9 [115.8, 178.3] ng/L), LDL-C (3.4 [2.6, 4.2] mmol/L), hs-CRP (3.6 [1.5, 11.2] mg/L), leukocytes (8.0 [6.8, 10.3] × 10^9/L), FPG (10.2 [7.5, 14.4] mmol/L), and HbA1c (10.2 [7.5, 14.4] %) were significantly higher in the T2DM-AIS group compared to the T2DM control group (Lp-PLA2: 102.1 [76.6, 121.9] ng/L, LDL-C: 3.2 [2.4, 3.7] mmol/L, hs-CRP: 2.4 [0.9, 5.2] mg/L, leukocytes: 7.0 [6.1, 8.1] × 10^9/L, FPG: 7.4 [6.1, 10.3] mmol/L, HbA1c: 7.0 [6.4, 8.2] %). In contrast, serum HDL-C levels (1.1 [0.9, 1.3] mmol/L) were significantly lower than those in the control group (1.3 [1.1, 1.5] mmol/L), which correlated with larger infarct size and greater neurological injury. ROC curve analysis indicated that the combined use of seven tests had an AUC of 0.906, with a sensitivity of 77.40% and a specificity of 95.30%.

**Conclusion:**

Monitoring serum levels of Lp-PLA2, LDL-C, HDL-C, hs-CRP, leukocyte count, FPG, and HbA1c provides a comprehensive assessment of cerebral infarction in T2DM patients with AIS and serves as auxiliary indicators for evaluating disease severity.

## 1 Introduction

Stroke is the leading cause of death and disability among adults in China. With accelerated aging of the population and rapid urbanization, the burden of stroke has increased dramatically, with ischemic stroke being the most prevalent type. Acute ischemic stroke (AIS) is defined by reduced blood supply to the brain due to arterial stenosis or occlusion, resulting in cerebral infarction, and is clinically referred to as “acute cerebral infarction” (1). Type 2 diabetes mellitus (T2DM) is a major high-risk factor for acute cerebral infarction (2, 3). In T2DM patients, insufficient insulin secretion or reduced insulin sensitivity leads to chronic hyperglycemia, which increases blood viscosity, promotes thrombus formation, and may result in vascular blockage, ultimately inducing cerebral infarction (3, 4). Additionally, prolonged hyperglycemia can lead to microvascular complications, such as retinopathy, nephropathy, and neuropathy, which may impair cerebral blood supply and further increase the risk of cerebral infarction (4) Lipoprotein-associated phospholipase A2 (Lp-PLA2) levels in plasma have been proposed as a biomarker for predicting AIS risk (5). Lp-PLA2-mediated cytokines can promote matrix metalloproteinase in atherosclerosis plaques and protease expression, and matrix metalloproteinases degrade the fibrous cap and collagen matrix and other components of the plaque, leading to plaque rupture and bleeding, which leads to the development of ischemic stroke (6, 7). Atherosclerosis (AS) contributes to cerebral artery stenosis and occlusion, leading to stroke (8, 9). Dyslipidemia, particularly elevated low-density lipoprotein cholesterol (LDL-C), plays a significant role in the development of AS (9). While high-density lipoprotein cholesterol (HDL-C) acts as a protective factor against AS (10). High-density lipoproteins (HDL) encompass a diverse group of lipoproteins characterized by an average size of 8-10 nanometers and a density ranging from 1.063 to 1.21 grams per milliliter. The fundamental composition of an HDL particle comprises a central core composed of esterified cholesterol, enveloped by a single layer consisting of phospholipids, free cholesterol, and apolipoproteins (apo) (11). Among these apolipoproteins, apoA-I and apoA-II are the predominant ones found in HDL particles (12). HDL-C is a key player in lipid metabolism and cardiovascular health. The quantity and quality of HDL can impact blood vessel health and the formation of atherosclerotic plaque, driven by oxidized LDL, a major factor in cardiovascular disease (13). Fasting plasma glucose (FPG) serves as an indicator of baseline glycemic state in T2DM patients with acute cerebral infarction, whereas glycated hemoglobin (HbA1c) reflects the average blood glucose level over the previous 120 days, as it is a stable compound formed by the covalent binding of glucose with the N-terminal valine of the hemoglobin β-chain. Persistent hyperglycemia in T2DM increases blood viscosity, promotes thrombogenesis, and damages vascular endothelium, thereby exacerbating AS (14). In type 2 diabetes Lp-PLA2 also exerts an anti-inflammatory action by degrading the pro-inflammatory mediator PAF and the oxidized phospholipids. Levels of PAF and oxidized phospholipids are elevated in inflammatory conditions leading to higher activity of Lp-PLA2 in order to degrade them. Therefore, Lp-PLA2 is a marker of inflammatory conditions but its role as an independent risk factor is uncertain (15). Following ischemic stroke onset, ischemia-induced vascular stimulation results in the release of numerous inflammatory factors, exacerbating cerebral injury. High sensitivity C-reactive protein (hs-CRP) is one such factor that promotes AS progression and increases stroke risk (16). This study aims to evaluate the clinical utility of Lp-PLA2, LDL-C, HDL-C, hs-CRP, leukocyte count, FPG, and HbA1c in assessing the risk and severity of AIS in T2DM patients.

## 2 Methods

### 2.1 Study participants

**Figure 1:**
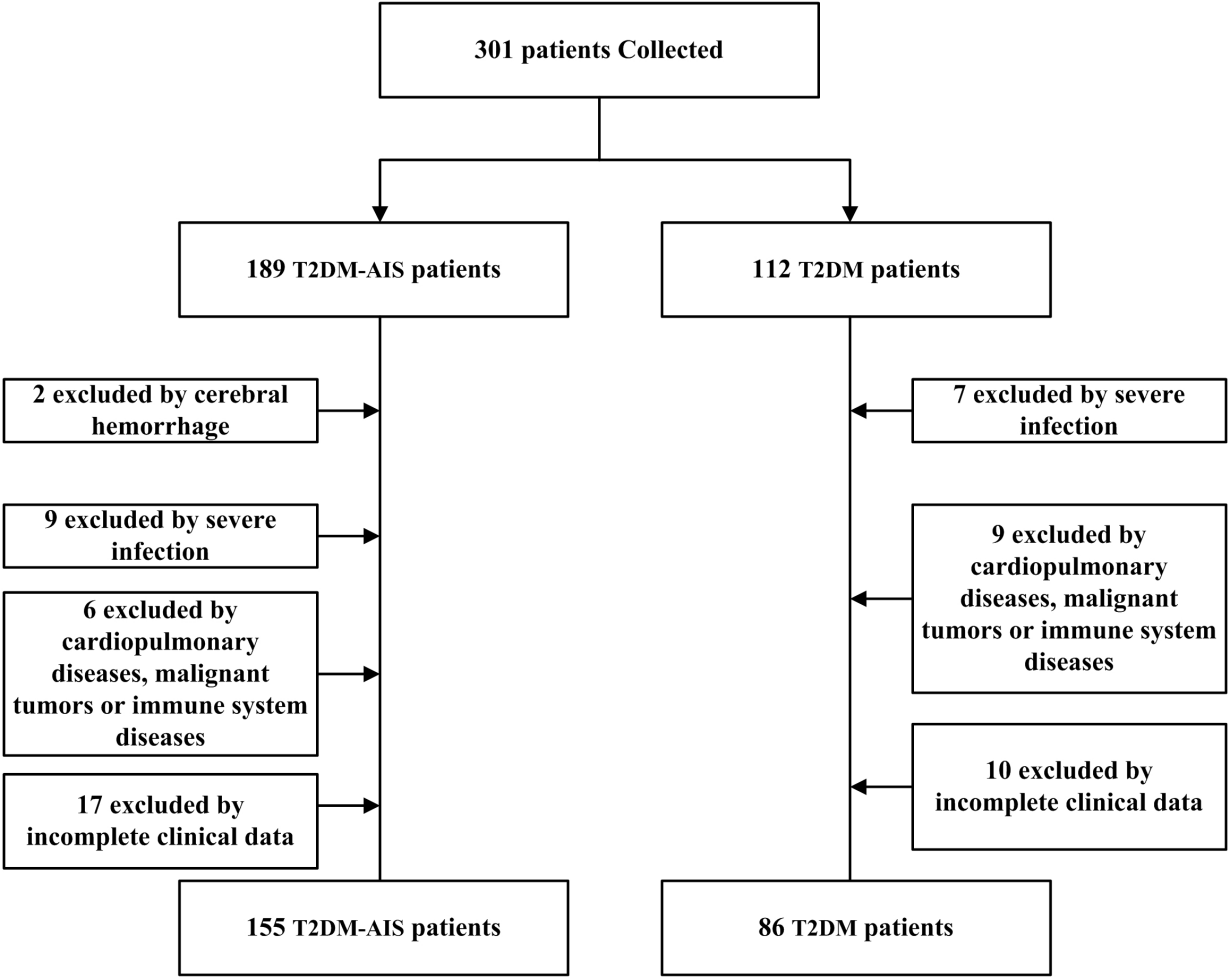
The flowchart of the study.

A total of 155 patients with type 2 diabetes mellitus (T2DM) complicated by acute ischemic stroke (AIS) admitted to the Neurology Department of the Second Affiliated Hospital of Shantou University Medical College from October 2023 to October 2024 were selected. The age range was 40 to 80 years, with an average age of 65.12 ± 7.94 years. There were 83 males and 72 females. Inclusion criteria were: (1) Diagnosis of T2DM in accordance with the 1999 World Health Organization (WHO) criteria for diabetes diagnosis (17); (2) AIS was definitively diagnosed by cranial CT or magnetic resonance imaging (MRI) (1), with the time from onset to hospital admission ≤ 72 hours; (3) No prior antiplatelet, anticoagulant, antithrombotic, or lipid-lowering therapy; (4) Normal liver and kidney function (Creatinine: 44-133μmol/L, ALT: 0-40 U/L). Exclusion criteria were: (1) Presence of bleeding tendencies; (2) Concurrent severe local or systemic infections; (3) Concurrent pulmonary diseases, cardiovascular conditions, malignant tumors, or immune system diseases. Additionally, 86 patients with T2DM without AIS admitted to the same department during the same period were selected as the control group, with an age range of 40 to 80 years and an average age of 64.67 ± 8.32 years. There were 39 males and 47 females. Inclusion criteria were: (1) Diagnosis of T2DM in accordance with the 1999 WHO criteria for diabetes diagnosis; (2) Normal liver and kidney function. Exclusion criteria were: (1) Concurrent severe local or systemic infections; (2) Concurrent cardiopulmonary diseases, malignant tumors, or immune system diseases (**Figure 1**). This study was approved by the Ethics Committee of the Second Affiliated Hospital of Shantou University Medical College.

### 2.2 Research methods

#### 2.2.1 Data collection and evaluation criteria

Clinical data, including Lp-PLA2, LDL-C, HDL-C, hs-CRP, leukocyte count, fasting plasma glucose (FPG), and glycated hemoglobin (HbA1c), were collected for both the T2DM-AIS group and the T2DM control group via the electronic medical records system. Lp-PLA2, LDL-C, HDL-C, hs-CRP, leukocyte count, and HbA1c were all data from the patient’s first serum measurement before medication after admission. Fasting plasma glucose (FPG) levels were determined at 6:00 AM on the second post-admission day after 8 hours of fasting. The National Institutes of Health Stroke Scale (NIHSS) scores were recorded at admission for the T2DM-AIS group to assess the degree of neurological impairment. Neurological impairment criteria (18) were defined as follows: NIHSS scores of 1-4 indicated mild impairment, 5-15 indicated moderate impairment, and > 15 indicated severe impairment. MRI results were also collected for the T2DM-AIS group, and infarct size was classified as small, medium, or large based on MRI findings. Infarct size classification (19) was as follows: a small infarct was defined as < 1/3 of the middle cerebral artery (MCA) territory affected; a medium infarct as 1/3 ≤ MCA territory < 2/3; and a large infarct as ≥ 2/3 of the MCA territory, with or without involvement of the anterior or posterior cerebral artery territories.

#### 2.2.2 Observation indicators

The serum levels of Lp-PLA2, LDL-C, HDL-C, hs-CRP, leukocyte count, FPG, and HbA1c were compared between the T2DM-AIS group and the T2DM control group. Based on infarct size and neurological impairment severity, T2DM-AIS patients were categorized into three infarct size groups (small, medium, and large; n = 95, 44, and 16, respectively) and three neurological impairment groups (mild, moderate, and severe; n = 89, 58, and 9, respectively). Differences in the above-mentioned indicators were compared between patients with small versus medium/large infarct sizes and between those with mild versus moderate/severe neurological impairment.

### 2.3 Statistical analysis

Data analysis and visualization were performed using R version 4.4.2. Continuous data were expressed as median (Q25, Q75), while categorical data were represented as frequency and percentage. The Shapiro-Wilk test and Levene’s test were used to assess normality and homogeneity of variance, respectively. For comparisons between two groups, an independent t-test was used if the data were normally distributed and had equal variance; otherwise, the Wilcoxon rank-sum test was applied. A p-value of < 0.05 was considered statistically significant. The receiver operating characteristic (ROC) curve and area under the curve (AUC) were used to evaluate the clinical utility of Lp-PLA2, LDL-C, HDL-C, hs-CRP, leukocyte count, FPG, and HbA1c, individually and in combination, for diagnosing AIS in T2DM patients.

## 3 Results

### 3.1 Comparison of baseline characteristics between T2DM-AIS group and T2DM control group

There were no statistically significant differences between the T2DM-AIS group and the T2DM control group in terms of gender, age, platelet count, smoking status, and blood pressure (all *P* > 0.05), indicating comparability between the two groups (**Table 1**).

**Table 1:** Comparison of baseline characteristics between the T2DM-AIS group and the T2DM control group.

Variables	Case (n=155)	Control (n=86)	*χ^2^*/Z	p*
Age, M (Q25, Q75)	65 (59, 72)	67 (59, 70)	-0.255	0.799
Gender, *n* (%)			1.178	0.278
Male	83 (53.5)	39 (45.3)		
Female	72 (46.5)	47 (54.7)		
Smoke, *n* (%)			2.446	0.118
Yes	117 (75.5)	56 (65.1)		
No	38 (24.5)	30 (34.9)		
Hypertension, *n* (%)			0.077	0.781
Yes	27 (17.4)	17 (19.8)		
No	128 (82.6)	69 (80.2)		
Plt, (10^9/L)	242.0 (189.9, 290.0)	224.0 (184.0, 261.5)	-1.942	0.052

### 3.2 Comparison of various indicators between T2DM-AIS group and T2DM control group

**Figure 2:**
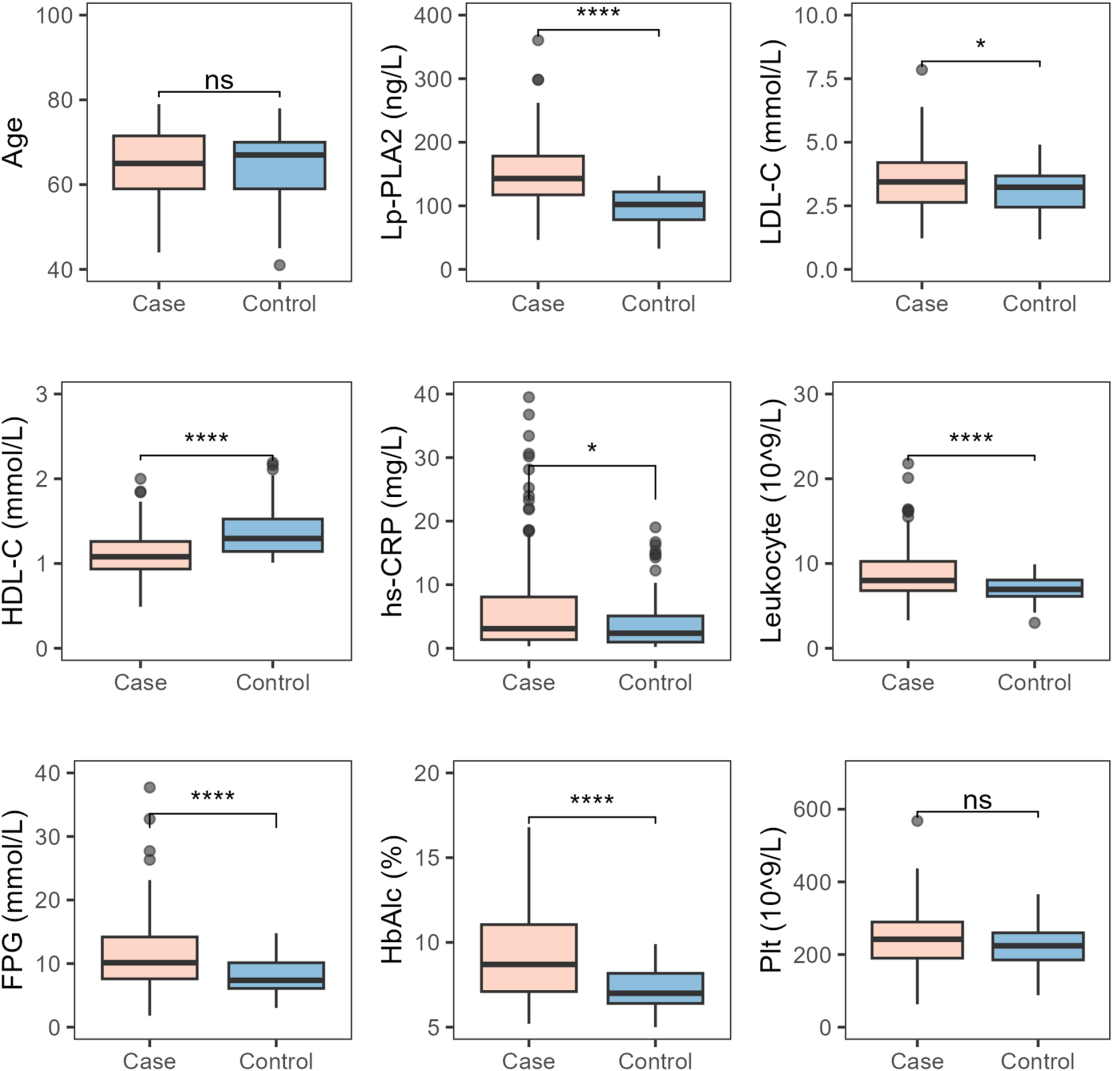
Comparison of various indicators between the two study groups. Case, T2DM-AIS group; Control, T2DM control group; HbA1c (%), Glycated Hemoglobin; LDL-C (mmol/L), Low-Density Lipoprotein Cholesterol; HDL-C (mmol/L), High-Density Lipoprotein Cholesterol; Lp-PLA2 (ng/L), Lipoprotein-Associated Phospholipase A2; hs-CRP (mg/L), High-Sensitivity C-Reactive Protein; Leukocyte (10^9/L), Leukocyte Count; FPG (mmol/L), Fasting Plasma Glucose; Plt (10^9/L), Platelet Count; ^*^P < 0.05; ^****^P < 0.0001; ns, no statistical significance.

There were no differences in age and platelet levels between the T2DM-AIS group and the T2DM control group. However, the T2DM-AIS group had significantly higher levels of serum Lp-PLA2 (*P* < 0.01), LDL-C (*P* < 0.05), hs-CRP (*P* < 0.05), leukocyte count (*P* < 0.01), FPG (*P* < 0.01), and HbA1c (*P* < 0.01) compared to the T2DM control group. In contrast, serum HDL-C levels were significantly lower in the T2DM-AIS group compared to the T2DM control group (*P* < 0.01) (**Table 2**, **Figure 2**).

**Table 2:** Comparison of quantitative characteristics between the T2DM-AIS group and the T2DM control group.

Variables	Case (n=155)	Control (n=86)	*χ^2^*/Z	p*
Lp-PLA2, *(ng/L)*	142.9 (115.8,178.3)	102.1 (76.6, 121.9)	-7.898	< 0.001
LDL-C, *(mmol/L)*	3.4 (2.6, 4.2)	3.2 (2.4, 3.7)	-2.025	0.043
HDL-C, *(mmol/L)*	1.1 (0.9, 1.3)	1.3 (1.1, 1.5)	-6.025	< 0.001
hs-CRP, *(mg/L)*	3.6 (1.5, 11.2)	2.4 (0.9, 5.2)	-3.019	0.003
Leukocyte, *(10^9/L)*	8.0 (6.8, 10.3)	7.0 (6.1,8.1)	-4.615	< 0.001
FPG, *(mmol/L)*	10.2 (7.5, 14.4)	7.4 (6.1, 10.3)	-4.922	< 0.001
HbA1c, *(%)*	8.7 (7.1, 11.1)	7.0 (6.4, 8.2)	-5.775	< 0.001

Lp-PLA2 (ng/L), Lipoprotein-Associated Phospholipase A2; LDL-C (mmol/L), Low-Density Lipoprotein Cholesterol; HDL-C (mmol/L), High-Density Lipoprotein Cholesterol; hs-CRP (mg/L), High-Sensitivity C-Reactive Protein; Leukocyte (10^9/L), Leukocyte Count; FPG (mmol/L), Fasting Plasma Glucose; HbA1c (%), Glycated Hemoglobin.

### 3.3 Comparative analysis of various indicators in T2DM-AIS patients with small vs. medium-to-large infarct area

**Figure 3:**
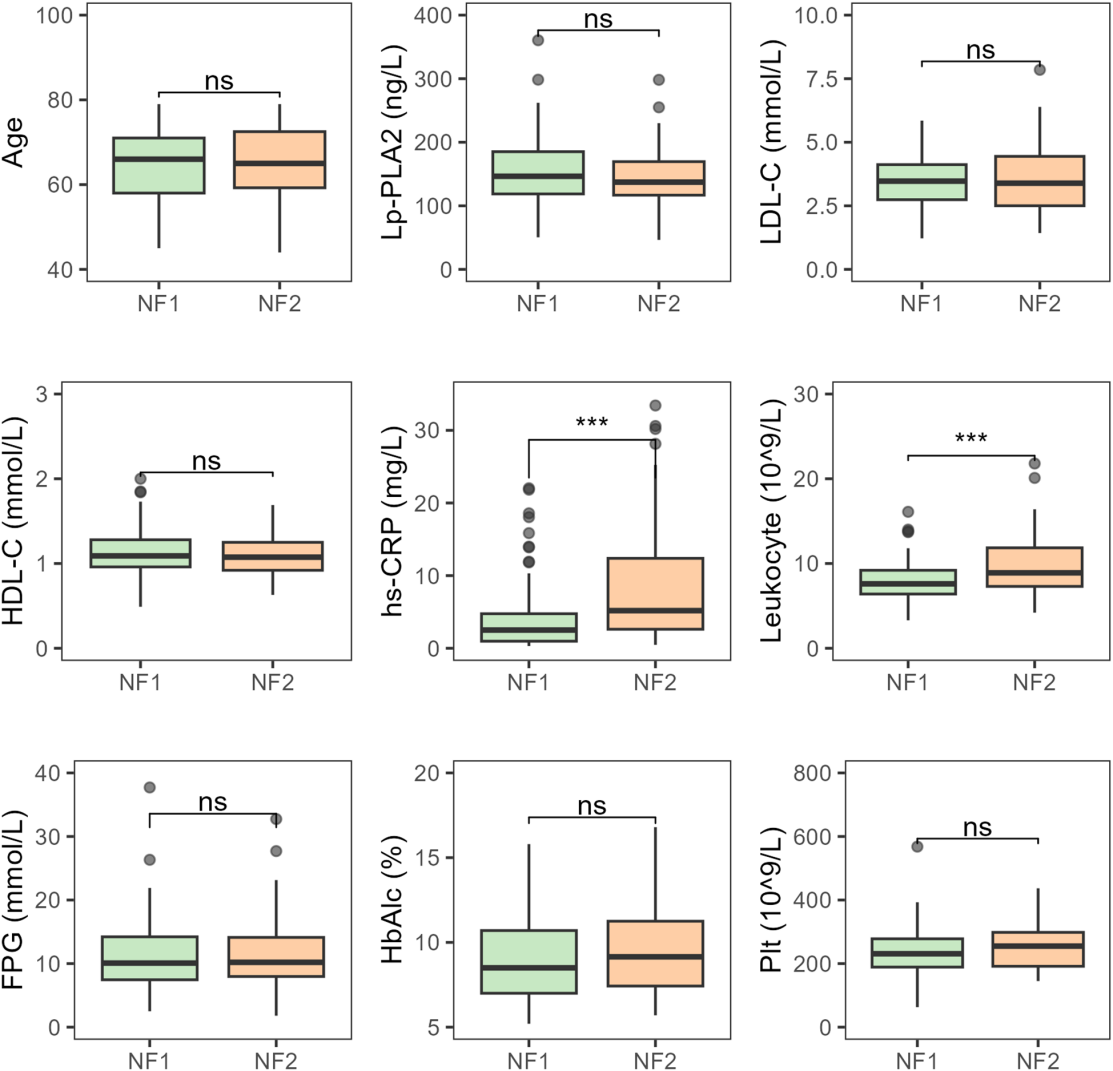
Comparative analysis of various indicators in T2DM-AIS patients with different infarct areas NF1, Small infarct area; NF2, Medium-to-large infarct area; HbA1c (%), Glycated Hemoglobin; LDL-C (mmol/L), Low-Density Lipoprotein Cholesterol; HDL-C (mmol/L), High-Density Lipoprotein Cholesterol; Lp-PLA2 (ng/L), Lipoprotein-Associated Phospholipase A2; hs-CRP (mg/L), High-Sensitivity C-Reactive Protein; Leukocyte (10^9/L), Leukocyte Count; FPG (mmol/L), Fasting Plasma glucose; Plt (10^9/L), Platelet Count; ^***^ *P* < 0.001; ns, no statistical significance.

There were no statistically significant differences between patients with small and medium-to-large infarct areas in terms of age, platelet count, HbA1c, LDL-C, HDL-C, and Lp-PLA2. However, hs-CRP and leukocyte counts showed significant differences between the two groups, with both hs-CRP and leukocyte levels increasing progressively as infarct area increased (both *P* < 0.001) (**Figure 3**).

### 3.4 Comparative analysis of various indicators in T2DM-AIS patients with different degrees of neurological deficit

**Figure 4:**
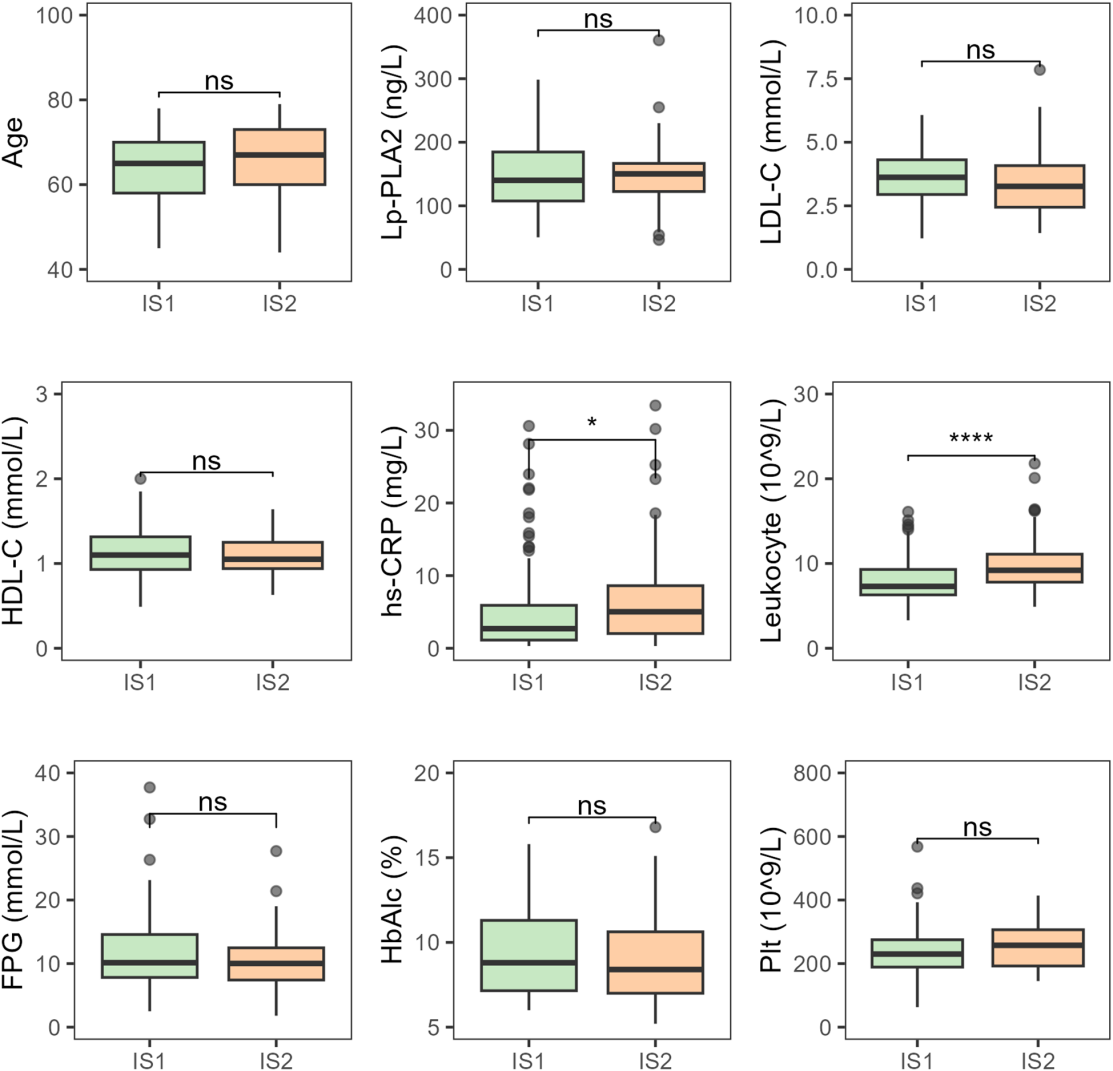
Comparative analysis of various indicators in T2DM-AIS patients with different degrees of neurological deficit. IS1, Mild neurological deficit; IS2, Moderate to severe neurological deficits; HbA1c (%), Glycated Hemoglobin; LDL-C (mmol/L), Low-Density Lipoprotein Cholesterol; HDL-C (mmol/L), High-Density Lipoprotein Cholesterol; Lp-PLA2 (ng/L), Lipoprotein-Associated Phospholipase A2; hs-CRP (mg/L), High-Sensitivity C-Reactive Protein; Leukocyte (10^9/L), Leukocyte Count; FPG (mmol/L), Fasting Plasma Glucose; Plt (10^9/L), Platelet Count; ns, no statistical significance; *P < 0.05; ^****^P < 0.0001.

There were no statistically significant differences between patients with mild and moderate-to-severe neurological deficits in terms of age, platelet count, HbA1c, LDL-C, HDL-C, and Lp-PLA2. However, hs-CRP (*P* < 0.05) and leukocyte counts (*P* < 0.001) showed significant differences between the two groups, with both hs-CRP and leukocyte levels increasing progressively as infarct size increased (**Figure 4**).

### 3.5 Effectiveness analysis of combined detection of seven indicators

**Figure 5:**
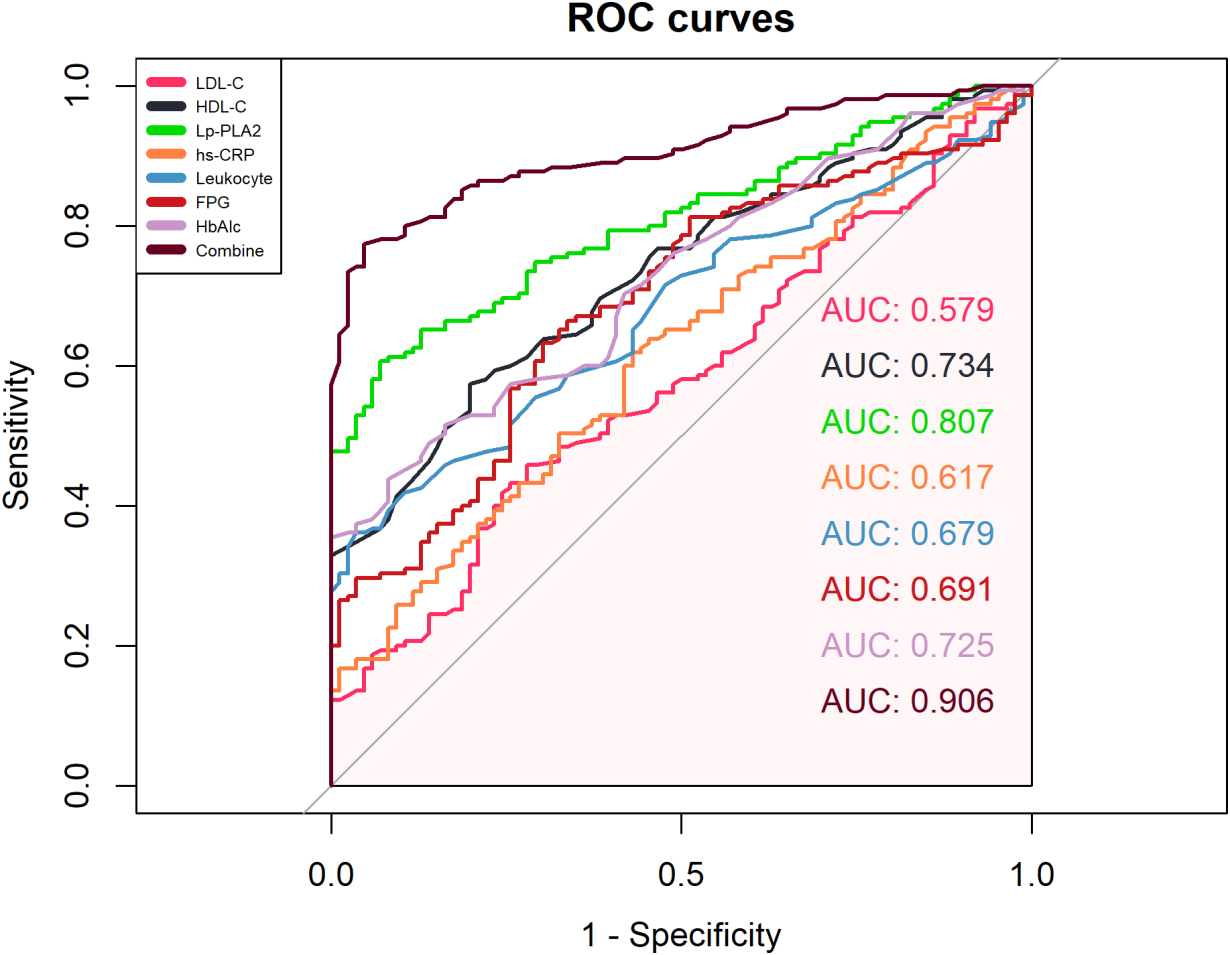
ROC curve analysis of each indicator.

ROC curve analysis showed that the combined detection of seven indicators had an AUC of 0.906, sensitivity of 77.40%, and specificity of 95.30% (**Figure 5**, **Table 3**).

**Table 3:** Performance of each indicator in detecting T2DM-AIS.

Variables	AUC	95%CI	Sensitivity/%	Specificity/%	Cutoff Value	Youden’s Index
Lp-PLA2	0.807	0.754 ~ 0.860	60.60	93.00	132.670	0.537
LDL-C	0.579	0.506 ~ 0.652	45.80	72.10	3.585	0.179
HDL-C	0.734	0.204 ~ 0.327	42.60	19.80	1.115	-0.377
hs-CRP	0.617	0.546 ~ 0.689	61.90	57.00	2.640	0.189
Leukocyte	0.679	0.614 ~ 0.745	36.10	96.50	9.150	0.326
FPG	0.691	0.624 ~ 0.759	63.20	69.80	9.040	0.330
HbA1c	0.725	0.662 ~ 0.787	43.90	91.90	9.150	0.357
Combine	0.906	0.870 ~ 0.942	77.40	95.30	0.738	0.728

Lp-PLA2 (ng/L), Lipoprotein-Associated Phospholipase A2; LDL-C (mmol/L), Low-Density Lipoprotein Cholesterol; HDL-C (mmol/L), High-Density Lipoprotein Cholesterol; hs-CRP (mg/L), High-Sensitivity C-Reactive Protein; Leukocyte (10^9/L), Leukocyte Count; FPG (mmol/L), Fasting Plasma Glucose; HbA1c (%), Glycated Hemoglobin; Plt (10^9/L), Platelet Count; AUC, Area under the curve.

## 4 Discussion

To date, AIS is a refractory disease that poses a serious threat to human health, with AS being the pathological basis of AIS and closely related to inflammatory responses (20). Inflammatory responses, degeneration, exudation, and thrombosis are the main pathogenic mechanisms of AS. Lp-PLA2, LDL-C, HDL-C, hs-CRP, Leukocyte, FPG, and HbA1c play significant roles in AS and are closely related to the occurrence and development of AIS. Therefore, exploring the levels of Lp-PLA2, LDL-C, HDL-C, hs-CRP, Leukocyte, FPG, and HbA1c in the serum of AIS patients and intervening actively holds important clinical value for the prevention and assessment of AIS patients with T2DM.

Lp-PLA2 is a member of the phospholipase family and is a vascular-specific inflammatory factor. In the bloodstream, 80% of Lp-PLA2 is bound to LDL-C. Also known as platelet-activating factor acetylhydrolase, Lp-PLA2 is primarily released by macrophages and neutrophils from AS plaques, playing an important role in the AS process by participating in plaque formation. Lp-PLA2 exhibits enzymatic activity by hydrolyzing oxidized phospholipids in low-density lipoproteins (LDL), resulting in the formation of lipid pro-inflammatory substances (such as oxidized free fatty acids and lysophosphatidylcholine), which produce multiple pro-AS effectors. Lp-PLA2 is considered a novel vascular-specific inflammatory factor and serves as an independent risk factor for cardiovascular and cerebrovascular events (21). Circulating Lp-PLA2 in the human body reflects the dynamic progression of vascular AS.Oei et al. (22) first identified Lp-PLA2 as a novel independent predictor of ischemic stroke through a cohort study of 1,820 participants in Rotterdam. LP-PLA2 is elevated in poorly-controlled patients compared to well-controlled diabetic patients (15). Monitoring Lp-PLA2 in patients with T2DM will be helpful to early warning and diagnosis of cardiovascular disease, and take on clinical significance in improving the prognosis of the disease (15, 23). Lp-PLA2 has been found to be abnormally up-regulated in type 2 diabetes, and its inhibitors have been used to treat the complications of type 2 diabetes with remarkable effects (15, 24–27). The results of this study showed that plasma Lp-PLA2 levels were significantly higher in the T2DM-AIS group compared to the control group (*P* < 0.01), indicating that Lp-PLA2 is abnormally overexpressed in AIS patients, which is consistent with the findings of Li et al. (28). Moreover, Lp-PLA2 levels in patients increased progressively with larger infarct areas and more severe neurological deficits, suggesting that Lp-PLA2 could assist in the prognostic evaluation of T2DM-AIS and serve as a predictive indicator.

Elevated LDL-C damages vascular endothelial and smooth muscle cells, triggering an inflammatory response in the vessel wall. LDL-C is recognized and phagocytosed by macrophages, forming foam cells, and the aggregation of foam cells is key to the formation and growth of AS plaques, thus leading to the development of AS (29). This study showed that plasma LDL-C levels were significantly higher in the T2DM-AIS group compared to the control group (*P* = 0.043 < 0.05), indicating abnormally elevated LDL-C in T2DM-AIS patients. Elevated LDL-C can also bind to Lp-PLA2, potentially accelerating AS progression. HDL-C is the only lipoprotein with anti-AS effects in the bloodstream (30). HDL-C controls the activation of monocytes and the proliferation of monocyte precursors, and inhibits the migration of macrophages and the oxidation of LDL-C. It also reduces the production of oxidized LDL-C, preventing endothelial cells from being affected by oxidative stress and inflammation, thus exerting protective effects against vascular AS. Elevated concentration of serum high‐density lipoprotein cholesterol (HDL‐C) protects against cardiovascular disease through a variety of mechanisms, including reverse cholesterol transport, anti‐inflammatory, antioxidant, and antithrombotic effects (31). Studies have confirmed that a stable negative correlation between HDL-C (high-density lipoprotein cholesterol) and stroke has been observed in different populations (32, 33). The results of this study show that plasma HDL-C levels were lower in the T2DM-AIS group compared to the control group (*P* < 0.01), indicating that plasma HDL-C is abnormally low in T2DM-AIS patients. Moreover, as infarct size increases and neurological deficits become more severe, HDL-C levels in the serum decrease.

Inflammation is a driving factor in the development of AS, and CRP is one of the important inflammatory markers. However, due to the relatively low sensitivity of CRP, hs-CRP has replaced CRP as a biomarker for predicting vascular events (34). The occurrence of AIS is closely related to elevated levels of hs-CRP. When hs-CRP levels increase, it can promote the rupture of unstable plaques, leading to the progression of vascular AS, activation of the complement system, suppression of the fibrinolytic system, and promotion of thrombosis, which contribute to the onset and development of stroke (16, 35). In the data of this study, the white blood cell (WBC) count in the T2DM-AIS group was higher than in the control group (*P* < 0.01), but the mean values of both groups were within the normal adult range (4×10^9/L to 10×10^9/L). In patients with T2DM-AIS during the acute phase, WBC counts were stress-induced and elevated (36), but this was less sensitive than hs-CRP (37). Therefore, this study analyzed both WBC count and hs-CRP. The results show that plasma hs-CRP levels in the T2DM-AIS group were significantly higher than in the control group (*P* = 0.03 < 0.05). The mean values for both groups were within the normal adult range (0.1-10 mg/L), indicating an abnormal high expression of hs-CRP in T2DM-AIS patients, which is consistent with previous literature (16). As the infarct area increases and neurological deficits worsen, hs-CRP levels rise, suggesting that hs-CRP may have certain significance in the occurrence, prognosis, and treatment assessment of T2DM-AIS patients.

Since FPG is less affected by diet, it is considered a more reliable measurement of blood glucose levels compared to random blood glucose levels (38). FPG is more reliable than admission blood glucose in evaluating glucose metabolism and predicting the 90-day prognosis in AIS patients. Cao et al.’s study demonstrated that FPG, independent of admission blood glucose, is a strong predictor associated with the prognosis of AIS patients undergoing intravenous thrombolysis (39). Among Chinese adults, higher FPG levels are independently associated with an increased risk of stroke, particularly in women, where serum FPG levels are significantly elevated in AIS patients (40). The results of this study show that FPG levels in the T2DM-AIS group were significantly higher than those in the control group (*P* < 0.01). Similar to LDL-C, FPG levels increased progressively with larger infarct areas and more severe neurological deficits.

HbA1c is considered the gold standard for assessing long-term glycemic control (41), while FPG may overlook the potential impacts of postprandial glucose levels and other significant factors (42). Patients with diabetes mellitus (DM) are more prone to atherosclerotic lesions, which often exhibit more inflammation infiltration (macrophages and T lymphocytes), larger necrotic core sizes, and more extensive atherosclerosis. Elevated impaired fasting glucose (IFG) can increase the relative risk of cardiovascular and cerebrovascular diseases, with high IFG positively correlated with the severity of arterial stenosis (43), highlighting the significant role of hyperglycemia in the progression of atherosclerosis. Increasing evidence suggests that hyperglycemia can induce excessive production of reactive oxygen species in cardiovascular cells’ mitochondria. Excessive reactive oxygen species can promote atherosclerosis by activating various pathways, including increased substrate conversion of aldose reductase (AKR1B1), increased formation of methylglyoxal (a precursor to advanced glycation end-products), and activation of protein kinase C isoforms β, δ, and θ. Hyperglycemia can also accelerate atherosclerosis by inducing endothelial dysfunction, reducing the bioavailability of nitric oxide, promoting vasoconstriction or a pre-thrombotic state, and enhancing the expression of nuclear factor-κ B (44). This study’s results show that plasma HbA1c levels were significantly higher in the T2DM-AIS group compared to the control group (*P* < 0.01), indicating poor glycemic control in the three months prior to onset, which can lead to increased platelet aggregation, blood viscosity, and blood thrombosis, accelerating pathological changes such as AS and exacerbating the progression of AIS. At the time of onset, the T2DM-AIS group showed significantly higher levels than the control group, suggesting that as the condition progresses, FPG increases, indicating that FPG levels are meaningful for assessing the occurrence, outcomes, and treatment efficacy of AIS in patients with T2DM. Stress-induced hyperglycemia is a marker of poor prognosis in AIS patients (45). Stress-induced hyperglycemia can reflect the extent of ischemic damage and result in worse clinical outcomes for stroke patients (46). The results of this study show that plasma FPG and HbA1c levels were significantly higher in the T2DM-AIS group compared to the control group (*P* < 0.01), with both increasing as the condition progressed. These biomarkers could serve as valuable indicators for assessing the onset, outcomes, and treatment efficacy of AIS in T2DM patients.

This comprehensive analytical approach offers significant reference value for predicting prognosis and disease progression in AIS patients with T2DM. Our study innovatively proposes the combined analysis of seven serum biomarkers as auxiliary indicators for evaluating disease severity in type 2 diabetes mellitus (T2DM) patients with acute ischemic stroke (AIS). Previous clinical investigations predominantly examined isolated or paired associations between these biomarkers and stroke outcomes, which limited their clinical applicability due to potential confounding factors. Through ROC curve analysis, it was found that the individual analysis of Lp-PLA2, LDL-C, HDL-C, Leukocyte, hs-CRP, FPG, and HbA1c levels had limited value in predicting the prognosis of AIS in T2DM, as multiple factors can interfere with the sensitivity and specificity of the assessment. However, the combined analysis of seven indicators resulted in an AUC of 0.906, which allows for the evaluation of the patient’s pathophysiological condition from multiple dimensions, such as endothelial cell injury, lipid metabolism abnormalities, vascular wall inflammation, and increased blood viscosity. This combined approach can provide valuable reference for predicting the prognosis and disease progression of AIS in T2DM patients. Therefore, exploring the levels of Lp-PLA2, LDL-C, HDL-C, hs-CRP, Leukocyte, FPG, and HbA1c in the serum of AIS patients and intervening actively holds important clinical value for the prevention and assessment of AIS patients with T2DM.

The current study has two limitations. First, after grouping patients with different infarct areas and different degrees of neurological impairment, we did not group the gender. This is because there is no gender ratio difference in the comparison of demographic information between the control group and the T2DM-AIS group. Therefore, we believe that gender is not a possible confounding factor between the two groups. Therefore, there is no further grouping study of the gender of patients with different infarct areas and different degrees of neurological impairment in the experimental design. Second, because we had to analyze seven indicators simultaneously, the final sample size was small. But the current results of this study offer a data basis for predicting prognosis and disease progression in AIS patients with T2DM. The study is not over, but a new starting point. In the next study, we will further increase the number of samples to elucidate the relationship between HDL-PLA2, LDL-C, HDL-C, hs-CRP, Leukocyte, FPG, and HbA1c in the serum of AIS patients and intervening actively holds important clinical value for the prevention and assessment of AIS patients with T2DM.

## 5 Conclusions

In summary, serum levels of Lp-PLA2, LDL-C, leukocyte count, hs-CRP, FPG, and HbA1c are significantly elevated, while HDL-C levels are markedly reduced in T2DM patients with AIS. These biomarkers can serve as auxiliary indicators for the assessment of disease severity in T2DM-AIS patients.

## Data Availability

The original contributions presented in the study are included in the article/**Supplementary Material**. Further inquiries can be directed to the corresponding author.
