# Supplementary material for: Clinical value of Lp-PLA2, LDL-C, HDL-C, hs-CRP, leukocyte, FPG and HbA1c in type 2 diabetes mellitus patients with acute ischemic stroke
Source: Front Endocrinol (Lausanne). 2025 Apr 17;16:1546961. doi: 10.3389/fendo.2025.1546961 (PMC12043486; doi:10.3389/fendo.2025.1546961)
Supplement: Supplementary file 1 [file Table1.docx]

**Supplementary data**

**TABLE S1.** Logistic regression analysis between the T2DM-AIS group and the T2DM control group

| **Variables** | **β（95%CI）** | **P value** |
| --- | --- | --- |
| HbAlc | 1.463(1.134-1.886) | 0.003 |
| LDL-C | 1.202(0.78-1.852) | 0.405 |
| HDL-C | 0.036(0.007-0.191) | 0.001 |
| Lp-PLA2 | 1.026(1.014-1.037) | 0.001 |
| hs-CRP | 1.01(0.973-1.05) | 0.592 |
| Leukocyte | 1.276(1.05-1.551) | 0.014 |
| FPG | 1.05(0.931-1.184) | 0.428 |
| Hypertension | 1.256(0.495-3.187) | 0.631 |
| Sex | 0.653(0.292-1.456) | 0.297 |
